# Gallic acid/hydroxypropyl-β-cyclodextrin complex: Improving solubility for application on *in vitro/ in vivo Candida albicans* biofilms

**DOI:** 10.1371/journal.pone.0181199

**Published:** 2017-07-11

**Authors:** Guilherme Rodrigues Teodoro, Aline Vidal Lacerda Gontijo, Aline Chiodi Borges, Márcia Hiromi Tanaka, Gabriela de Morais Gouvêa Lima, Marcos José Salvador, Cristiane Yumi Koga-Ito

**Affiliations:** 1 Oral Biopathology Graduate Program, São José dos Campos Institute of Science and Technology, Universidade Estadual Paulista/UNESP, São José dos Campos, SP, Brazil; 2 Departament of Environmental Engineering, São José dos Campos Institute of Science and Technology, Universidade Estadual Paulista/UNESP, São José dos Campos, SP, Brazil; 3 Department of Plant Biology, Institute of Biology, Universidade Estadual de Campinas/UNICAMP, Campinas, SP, Brazil; Yonsei University, REPUBLIC OF KOREA

## Abstract

The aim of this study was to increase the solubility of gallic acid (GA) for the treatment of *Candida albicans* biofilm, which is very difficult to treat and requires high drug concentrations. Cyclodextrins (CDs) were used for this purpose. Complexes were evaluated by phase-solubility studies, prepared by spray drying and characterized by drug loading, scanning electron microscopy (SEM) and differential scanning calorimetry (DSC). The complexes were tested on *C*. *albicans* biofilm using *in vitro* and *in vivo* models. HPβCD formed soluble inclusion complexes with GA. The percentage of GA in GA/HPβCD was 10.8 ± 0.01%. The SEM and DSC analyses confirmed the formation of inclusion complexes. GA/HPβCD maintained the antimicrobial activity of the pure GA. GA/HPβCD was effective on *C*. *albicans* biofilms of 24 and 48h. The *in vivo* results showed an anti-inflammatory activity of GA/HPβCD with no difference in invading hypha counting among the groups. This study encourages the development of new antifungal agents.

## Introduction

Although *Candida* genus commensally colonizes some mucosal surfaces, certain species can, at some point, produce candidoses in humans, and are thus considered an opportunistic pathogen [1]. Epidemiological data showed that *Candida* spp. are frequently associated with nosocomial systemic infections, with mortality rate of patients as high as 40% [2]. *C*. *albicans* is the most common *Candida* species causing systemic infections worldwide [2]. Its major virulence factor is its ability to form biofilms [3].

*C*. *albicans* biofilms are very difficult to treat. High drug concentrations are required to reduce their formation. For instance, in order to reach antibiofilm effect, concentrations 5 to 8 times higher than the minimal inhibitory concentration for planktonic cells are needed [4]. A previous study using scanning electron microscopy showed that a concentration 11 times the MIC for amphotericin B was not able to disrupt *C*. *albicans* biofilm [4]. Additionally, alternatives for the treatment of *C*. *albicans* biofilms in catheters, such as lock therapy, also require quite high drug concentrations (from 100 to 1000 times the MIC) [5, 6].

Nonetheless, problems of aqueous solubility start to appear at high drug concentrations. Cyclodextrins (CDs) may help to overcome this problem [7]. Because of their cyclic structure, which presents a hydrophilic outer surface and a lipophilic central cavity, CDs are able to form inclusion complexes with certain lipophilic drugs increasing their aqueous solubility [8]. Some antifungals, such as itraconazole and voriconazole, have already been complexed with CDs and marketed worldwide [9, 10]. Beta-cyclodextrin (βCD) is a natural type of CD which is frequently used [9]. However, its aqueous solubility can be rather limited [9]. 2-hydroxypropyl-beta-cyclodextrin (HPβCD), which is a βCD derivative, improves its aqueous solubility and presents thus pharmaceutical interest [9, 11].

Gallic acid (GA), a natural bioactive compound, showed promising antifungal activity on planktonic *C*. *albicans* in a previous study by our group [12]. However, this compound presents limited solubility, which could compromise the treatment of *C*. *albicans* biofilms. Therefore, the aim of this study was to increase the GA solubility using CDs. For this purpose, we have first investigated whether CDs (βCD and HPβCD) form soluble complexes with GA by phase-solubility studies. Drug–cyclodextrin complexes were prepared by spray drying and characterized by drug loading, differential scanning calorimetry (DSC) and scanning electron microscopy (SEM). Finally, the effect of complexes was tested on *in vitro* and *in vivo C*. *albicans* biofilms.

## Material and methods

### Materials

GA was purchased from Santa Cruz Biotechnology (Santa Cruz, USA). βCD and HPβCD were obtained from Sigma Aldrich (St. Louis, USA). All other reagents and solvents were of analytical grade.

### Phase solubility study

The effect of complexation with βCD and HPβCD on the solubility of GA was assessed. The phase solubility diagram was used in order to evaluate the solubility of GA in the presence of CDs according to Higuchi & Connors [13]. The amount of GA was kept in excess and mixed up with different concentrations of βCD (0–16 mM) and HPβCD (0–144 mM). The dispersions were kept at room temperature for 72 h on a magnetic stirrer, and were then filtered using a membrane of 0.45 μm. The dispersions were assayed for GA content by spectrophotometry at 260 nm (Biotek, Vermont, USA).

The phase solubility diagram was constructed by plotting concentrations of dissolved GA against CD concentrations. The complexation efficiency (CE) was determined by the slope of the phase solubility diagram, CE = slope/(1-slope) (Equation 1) [14]. The proportion between GA (drug, D) and HPβCD (cyclodextrin, CD) was obtained according to the equation D:CD = 1:(1+1/CE) (Equation 2), described by [15].

### Preparation of complexes

Solid systems were prepared by spray drying (Büchi Mini Spray Drying B 290, Flawil, Switzerland). The parameters adopted were: inlet air temperature at 120°C, outlet air temperature at 70°C, sample pump at 18%, aspirator at 80% and nozzle cleaner.

The inclusion complexes were obtained in the proportion of 1:1 molar. GA (0.616 g) was dissolved in 6 ml of methanol and HPβCD (5 g) was dissolved in 20 mL of distilled water. The solutions were mixed and then put into the spray dryer. After drying, the percentage of GA was detected by high-performance liquid chromatography (HPLC). The encapsulation efficiency (EE) was determined using Equation 3: EE = (C_GA encapsulated_ /C_GA initially added_) × 100; where C_GA encapsulated_ is the concentration of GA encapsulated determined by HPLC and C_GA initially added_ is the concentration of GA initially added.

### Characterization of GA/HPβCD spray-dried complexes

#### Analysis of GA in GA/HPβCD spray-dried complexes by high-performance liquid chromatography (HPLC)

Analysis of GA was performed by HPLC according to previously described methodology [16]. The equipment was composed by a Shimadzu SIL-20A HT auto-injector (Shimadzu Corporation, Kyoto, Japan), a LC-20AT pump (Shimadzu Corporation, Kyoto, Japan) and a SPD-M20A detector (Shimadzu Corporation, Kyoto, Japan). Chromatography was performed with a LiChrospher® 100 RP-18e column (5 μm, 250 x 4 mm) and an RP-18 pre-column cartridge. The mobile phase was composed by 97.5% of water with 2.5M ortophosphoric acid and 2.5% of acetonitrile (v/v). Flow rate was 1ml/min. An aliquot of 40μL of the sample was injected and analyses were performed using the LC real time analysis software. A retention time of 9 min was observed for GA. Nine points of the standard solutions were prepared for determination of a calibration curve with concentrations between 0.5–200 μg/ml. Three levels of control solution (low, intermediate and high) were also prepared. Analytical curves were adjusted by linear regression using 1/X^2^ as a weighing factor, where X was the GA concentration. Standard calibration curves were linear with R^2^ higher than 0.99. The accuracy and precision of the quantitative analysis methodology were lower than 15% for all levels of control (intraday and interday).

#### Differential scanning calorimetry (DSC)

Powder samples were analyzed by DSC (DSC 2910, New Castle, USA). Aliquots of 2 to 4 mg of GA/HPβCD spray-dried complexes and physical mixtures were analyzed. Also, analyses of GA and HPβCD raw materials were performed. The samples were heated from 25°C to 300°C at a rate of 10°C/min. The same GA mass proportion (11%) was used for the physical mixture and the GA/HPβCD.

#### Scanning electron microscopy study

Aliquots of the physical mixture, GA/HPβCD inclusion complexes, GA and HPβCD raw materials were analyzed by using scanning electron microscope (Leo 440i, LEO Electron Microscopy, Oxford, UK). Samples were fixed to stubs with the aid of carbon adhesive pad and submitted to gold metallization for 180 s. Samples were subsequently analyzed in order to detect changes in the surface morphology. The same GA mass proportion (11%) was used for the physical mixture and the GA/HPβCD.

### Antifungal activity of GA

#### Determination of minimum inhibitory concentration (MIC) values

Minimum inhibitory concentration (MIC) of pure GA was determined using 27 *C*. *albicans* clinical isolates and two references strains ATCC 18804 and SC 5314. Clinical isolates were obtained from oral lesions of candidiasis in a previous study [17]. The protocol of this study was approved by the Local Ethical Committee (#CAAE 085729127.0000.0077), Comissão Nacional de Ética em Pesquisa (CONEP).

Samples were stored in BHI broth supplemented with glycerol at 20% at -80°C. Prior to the experiments the microorganisms were plated on Sabouraud dextrose agar. Plates were incubated at 37°C for 24 h and suspensions were prepared in sterile saline solution. Suspensions were standardized spectrophotometrically. Broth microdilution methodology with RPMI 1640 buffered with 3-(N-morpholino)propanesulfonic acid (pH 7.0) was performed according to the National Committee for Clinical Laboratory [18]. After incubation for 24 h at 37°C, fungal growth was analyzed and compared with positive control. The lowest concentration able to inhibit the fungal growth in relation to control was considered as MIC. The concentration required to inhibit the growth in 50% and 90% of samples were labelled MIC_50_ and MIC_90_, respectively.

#### Antifungal activity of GA/ HPβCD spray-dried complexes

The value of MIC for GA/HPβCD inclusion complexes was obtained using the same methodology described for pure GA. *C*. *albicans* ATCC 18804 and SC 5314 were tested.

#### Effect of GA and GA/HPβCD on *C*. *albicans* biofilms

The antibiofilm effect of GA in its pure and GA/HPβCD spray-dried forms was determined. Pure GA at 2 and 4 times the MIC_90_ (5 mg/mL) were tested. The latter was insoluble in water and GA/HPβCD inclusion complexes were tested. The methodology was based on Cheng et al. [19], with modifications. *C*. *albicans* ATCC 18804 and SC 5314 and 3 clinical isolates were used in these experiments.

Polystyrene specimens (50mm^2^) were previously sterilized by UV radiation for 30 min. Fungal inoculum was obtained in sterile saline solution (NaCl 0.9%) and adjusted spectrophotometrically to 10^7^ cells/ml.

Aliquots of 20 μl of inoculum were added to 200 μl of RPMI supplemented with 2% glucose in 96-well plates. Specimens were added to each well immersed in the culture medium. Plates were incubated at 37°C under agitation (80 rpm) for 120 min for pre-adherence phase. The specimens were then washed with sterile saline solution and culture medium was refreshed. For 48h biofilms, the culture medium was also refreshed after 24h of incubation.

Biofilms were then exposed to 2 and 4 times MIC_90_ of GA and GA/HPβCD, respectively, for 5 min at room temperature. After this, the specimens were washed with sterile saline solution and transferred to tubes containing 1 ml of saline solution. For biofilm disruption, tubes were vortexed for 60 sec and then sonicated (2 pulses with 15 s interval, amplitude of 40 and 15W). Tubes were maintained in ice to avoid heating. Final suspensions were serially diluted in saline solution and plated on Sabouraud agar. Plates were incubated at 37°C for 48 h and values of colony forming units per specimen were calculated. Negative controls were immersed in saline solution at the same conditions. Positive controls were exposed to solution of amphotericin B at 2 μg ml^-1^.

#### Effect of GA/HPβCD for the treatment of oral candidiasis induced in rat model

Besides the biofilm, an *in vivo* experiment was performed to verify the anticandidal effect of GA/HPβCD inclusion complexes.

The *in vivo* assay was based on Nett et al. [20] and Okada et al. [21] with modifications, and was approved by ethical standards of the institution where the study was conducted (03/11- Comissão de Ética no Uso de Animal, CEUA/ICT-CJSC-UNESP). Before all clinical procedures, animals were anesthesized with xylazine/ketamine 2% aqueous solution (1:0.5 ml/100 g of body weight). Fifteen male rats (*Rattus norvegicus* Wistar) were divided in 3 groups: Control (treated with saline solution), Nystatin (100,000 UI/ml—positive control) and GA/HPβCD inclusion complexes. The animals were 3-month old and weighted approximately 350 g. During the experiment, animals were kept in cages with water and food *ad libitum*.

Before *C*. *albicans* (ATCC18804) inoculation, animals were treated orally with aztreonam (50mg/Kg) for 48 hours and then immunosuppressed with intradermic prednisolone (100 mg/Kg). Immunosuppression was done every other day until euthanasia. One day after the first prednisolone shot, *C*. *albicans* inoculum (10^6^ cells/ml) was applied with a swab on the palate surface for 3 min in a total of 2 applications with a 24-hour interspace. Twenty-four hours after the second inoculation, 100 μl of the antifungal or saline solution was administered orally, twice a day during 4 days, and then the rats were euthanized with excessive anesthetic solution.

Palate mucosa was removed and kept in 4% formalin for 48 hs, washed and kept in 70% ethanol for 24 h until processing and embedding. 5 μm sections were then placed in glass slides and stained with Hematoxilin and Eosin (HE) or Periodic acid-Shiff (PAS) to evaluate the inflammation process, epithelial alterations and hyphae presence and distribution, respectively. Two histologic sections in different depths were analyzed, per animal, and described according to the group. In PAS stained slides, a total of 21 histologic fields were analysed per section, totalizing 42 fields/tongue, using 40X objective lens (Zeiss). Hypha count was performed and a score was given to each field as follows: 0: hyphae absence; 1: 1–5 hyphae; 2: 6–15 hyphae; 3: 16–50 hyphae; and 4: more than 50 hyphae [22].

### Analysis of data

The obtained data were evaluated by statistical analysis at the level of significance of 5% (GraphPad Software Inc. San Diego, USA). The D'Agostino & Pearson omnibus normality test was applied in order to evaluate the distribution of biofilm data. The analysis showed that the distribution was not normal and thus the nonparametric Kruskal-Wallis test and Dunn's Multiple Comparison post hoc test were used to compare the treatment groups against the negative control. For the *in vivo* evaluation, a median of the scores obtained from the 42 histologic fields was determined per animal. Kruskal-Wallis and Dunn tests were applied to review scores (*p* ≤ 0.05).

## Results and discussion

### Phase solubility study

**Fig 1:**
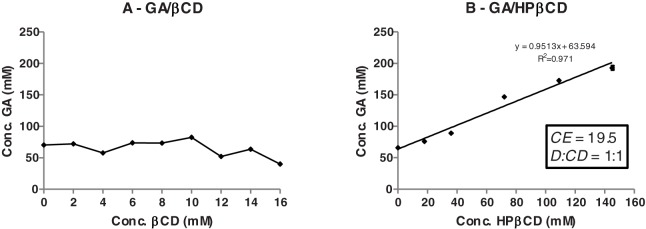
Phase solubility diagrams. Gallic acid (GA) in aqueous solutions of βCD (A) and HPβCD (B). CE is the complexation efficiency and D:CD is the molar ratio between GA and HPβCD.

For GA/βCD complexes, increasing in the βCD concentration led to a decrease of the GA solubility (Fig 1A). This curve profile is an indicative for the formation of insoluble complexes. Conversely, the curve for GA/HPβCD complexes is characterized by a linear increase in the GA solubility, which suggests the formation of soluble inclusion complexes. Similar results were previously obtained in studies with triclosan [23, 24]. Although βCD is hydrophilic, its aqueous solubility is rather limited (18.5 mg/mL), most probably due to relatively strong binding of the CD molecules in the crystal form [25]. Due to the low solubility of GA/βCD, further experiments and analyses were performed only with GA/HPβCD complexes.

HPβCD is a βCD derivative, with substitutions of the hydroxy groups by hydroxypropyl, resulting in a dramatic enhancement of its solubility (> 600 mg/mL) [25]. In general, the aqueous-soluble CD derivatives produce soluble complexes, whereas the less soluble CDs, such as βCD, frequently produce insoluble or poorly soluble complexes [25, 26].

The solubility curve for complexation of GA with HPβCD was well fitted by a linear function (R^2^ = 0.971) (Fig 1B). It was observed that 145 mM of HPβCD increased approximately three times the water solubility of GA. The complexation efficiency (CE) for GA/HPβCD calculated from equation 1 was 19.5 (Fig 1B). This equation considers the slope (63.594) of the phase diagram obtained by the linear equation. With the CE result, the molar ratio (D:CD) between GA and HPβCD can be determined (Equation 2). As shown in Fig 1B, the D:CD was equal to 1:1, which means that one molecule of GA binds to one molecule of HPβCD. Theoretically, considering the molar mass of GA and HPβCD (170.2 g/mol and 1,380 g/mol, respectively) 11g of GA should be found in 100g of GA/HPβCD complexes (11%). Previous studies have successfully encapsulated GA with HPβCD by spray drying technique with different parameters from this present study [27] and by electrospinning [28].

### Characterization of GA/HPβCD spray-dried complexes

#### Analysis of GA in GA/HPβCD spray-dried complexes by high-performance liquid chromatography (HPLC)

The percentage of GA present in GA/HPβCD, measured by HPLC, was 10.8 ± 0.01%. This experimental result is in accordance with the prediction above, which estimated a value of 11% of GA present in GA/HPβCD. The percentage of GA in GA/HPβCD after 24h under magnetic stirring at room temperature was 9.5 ± 2.6%, which suggest stability of GA after 24h in water. The chosen technique in this present study (spray drying) is known as one of the most effective for drug inclusion into CDs [29]. The encapsulation efficiency (EE) was almost 100% (Equation 3). A previous study [27] using the same technique (spray drying) but different parameters showed a lower EE (< 90%) for the complexation between GA and HPβCD. High EE indicates low waste of raw material, which is economically interesting.

#### Differential scanning calorimetry (DSC)

**Fig 2:**
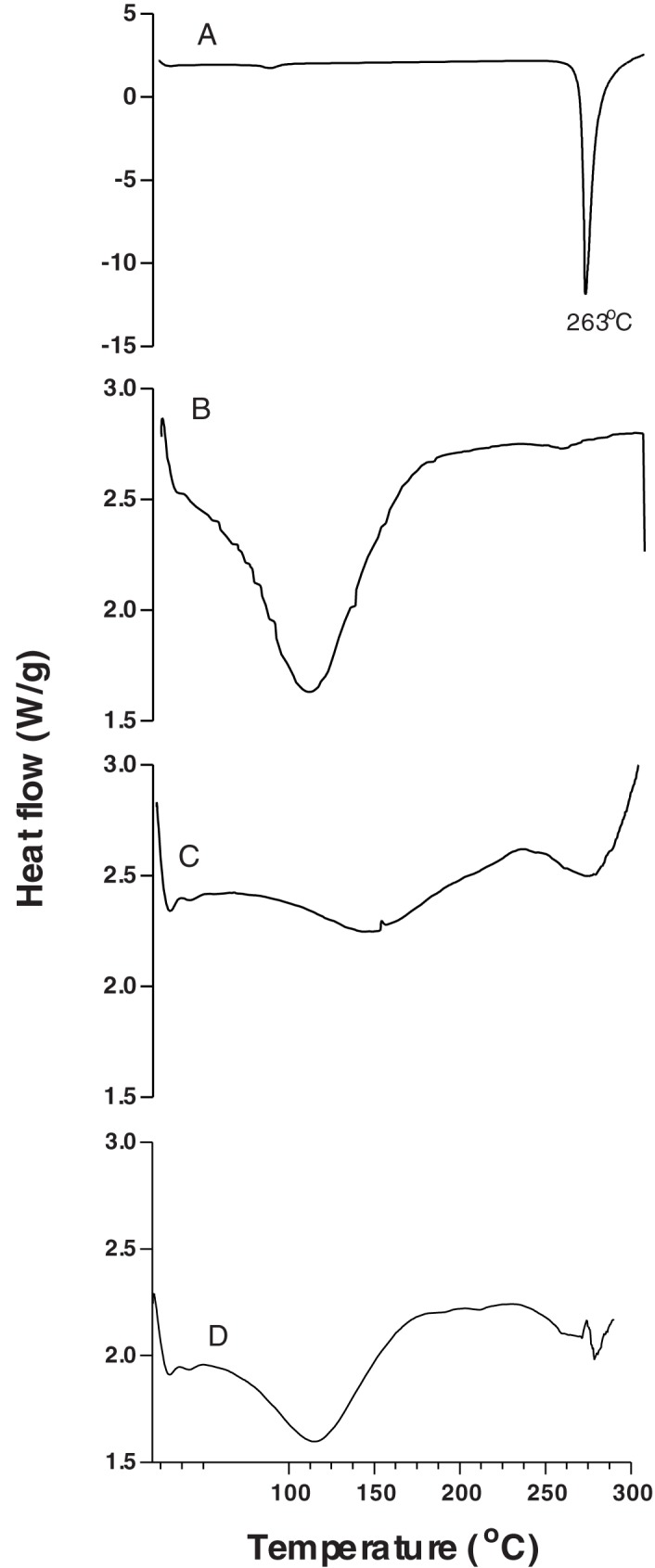
DSC thermograms. Gallic acid (GA) raw material (A), HPβCD (B), physical mixture between GA and HPβCD (C) and GA/HPβCD spray-dried particle (D).

The DSC thermogram of GA (Fig 2, curve A) showed that its melting endothermic peak was 262^°^C, confirming the crystalline conformation of the starting material. The thermogram of GA in this present study was quite similar to the one in a previous work, which showed melting endotherm peak of GA at 267^°^C [27].

The thermogram of HPβCD (Fig 2, curve B) showed a single endothermic peak centered at approximately 110^°^C, which is associated with the dehydratation of HPβCD. This present result is in accordance with previous work, which showed a first peak of temperature for HPβCD of approximately 100^°^C corresponding to the loss of water molecules from the cavity [30].

Moreover, the thermogram of the GA/HPβCD spray-dried complexes (curve D) showed substantial changes in comparison to the physical mixture (curve C). GA and HPβCD in the physical mixture, where no chemical interactions are expected, are in the same mass proportion as in GA/HPβCD spray-dried. Therefore, the changes between curves C and D can be explained by an interaction between GA and HPβCD in the GA/HPβCD spray-dried complexes, and hence can be an indicative of GA inclusion by HPβCD.

#### Scanning electron microscopy (SEM) study

**Fig 3:**
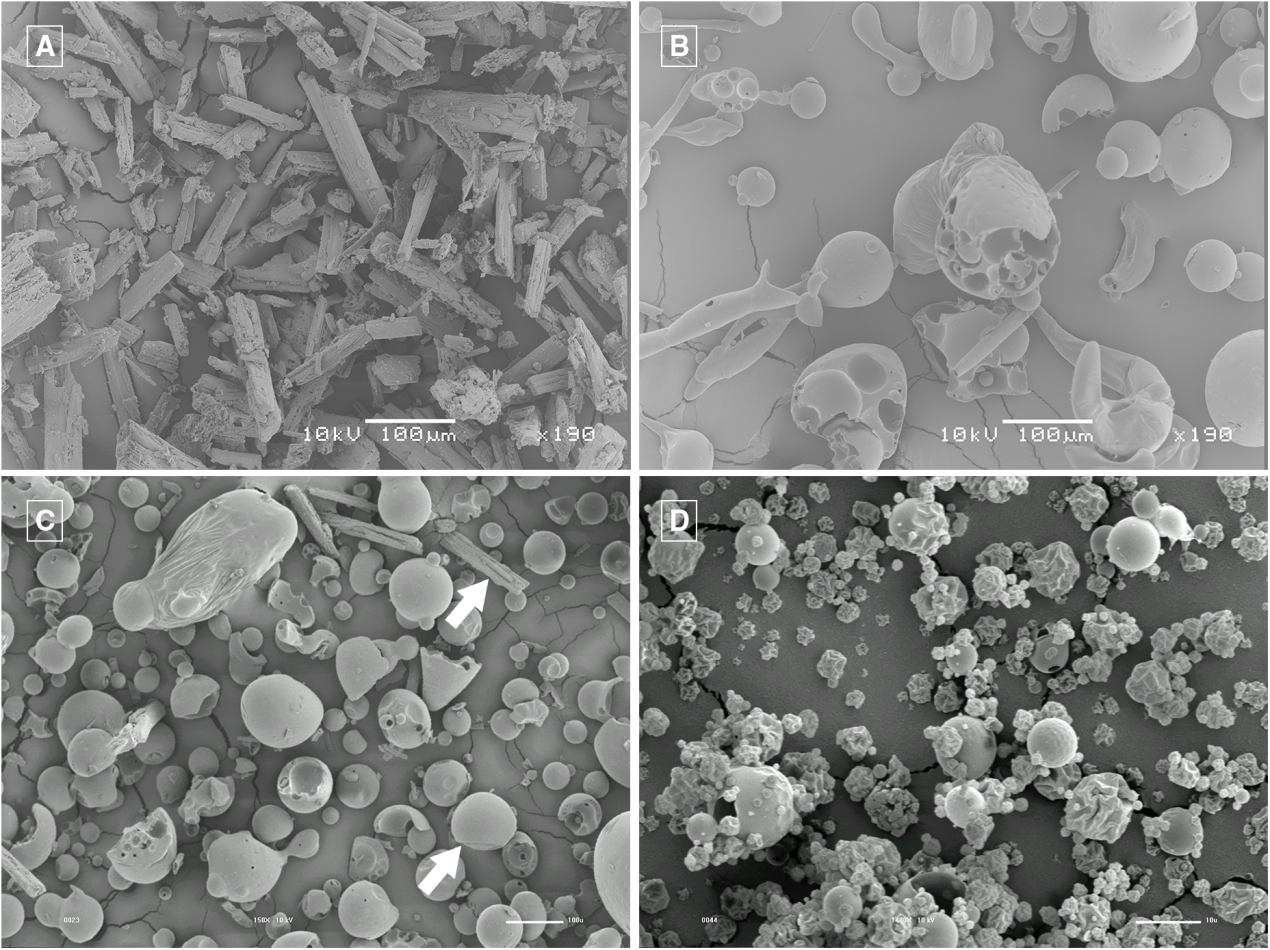
SEM micrographs. Gallic acid (GA) 190X magnification (A), HPβCD 190X magnification (B), physical mixture of GA and HPβCD 150X magnification (C) and GA/HPβCD spray-dried microparticles, 2,000X magnification (D). In Fig C the upper arrow points out to the GA morphology and the lower arrow points out to the HPβCD one.

The spray-dried microparticles (Fig 3D) exhibited a different morphology from the GA and HPβCD raw materials (Fig 3A and 3B), and this conformal modification suggeststhat there was an encapsulation of GA by HPβCD. As expected, the equimolar physical mixture (Fig 3C) presented morphological characteristic of GA and HPβCD in their pure forms. The GA presented a flat column form whereas the HPβCD presented a spherical form.

The combination of the results from different techniques (such as phase solubility studies, quantitative analysis of GA in GA/HPβCD spray-dried complexes by HPLC, DSC and SEM images) are indicative that there was a formation of GA/HPβCD complexes.

### Antifungal activity of GA

#### Determination of minimum inhibitory concentration (MIC) values

The increasing resistance of *C*. *albicans* to conventional antifungals indicates the need of new active molecules. Plants are sources of active molecules with potential antimicrobial activity [31, 32]. GA is a phenolic molecule compound found in many plant species with antimicrobial activity [12, 33, 34]. In the present study, GA presented MIC values of 0.625 and 1.25 mg/mL for *C*. *albicans* ATCC 18804 and SC 5314, respectively. For clinical strains the MIC_50_ and MIC_90_ for GA were respectively 2.5 e 5.0 mg/mL. The MIC values for GA varied thus between 0.625 and 5.0 mg/mL using different clinical and standard strains of *C*. *albicans*. The inclusion of clinical strains aimed to evaluate the intra-species variability in susceptibility. Therefore, MIC was considered 5 mg/mL for further studies with GA.

#### Antifungal activity of GA/HPβCD spray-dried complexes

GA/HPβCD spray-dried microparticles also presented MIC values equivalent to 0.625 and 1.25 mg/mL of GA for *C*. *albicans* ATCC 18804 and SC 5314 respectively, suggesting that the complexation process does not alter the antifungal activity of GA for planktonic *C*. *albicans* and maintain its antimicrobial activities, as previously observed in studies with Posaconazole/HPβCD [35]. Maintain the pharmaceutical effect of a drug while develop a novel drug formulation is the foundation of other applications [35]. It is not surprising that the MIC values were not changed, since at these concentrations (0.625 and 1.25 mg/mL), GA presents a good water solubility. Nonetheless, Tewes *et al*. [36] attested that when a fraction of antimicrobial agent is complexed with CDs, they should improve its activity, because only the free fraction of antimicrobial should be active.

#### Effect of GA and GA/HPβCD on *C*. *albicans* biofilms

**Fig 4:**
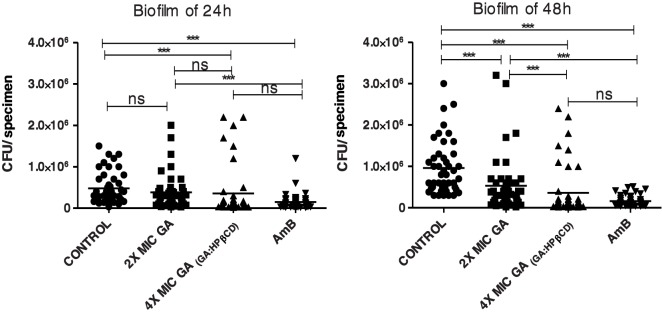
**Effect of galic acid (GA) on *C*. *albicans* biofilm of 24 h (A) and 48h (B).**Biofilms were exposed to 2 and 4 times the MIC (10 and 20 mg/mL) of GA for 5 min or to amphotericin B (AmB, 2 μg ml^-1^). Four times the MIC of GA (20 mg/mL) was insoluble in water and GA/HPβCD spray-dried microparticles were tested. Control refers to negative control. Each point represents the mean of the data from three independent experiments (n = 45); bars represent the standard deviation (SD). Kruskal-Wallis test and Dunn's Multiple Comparison post hoc test were used to compare the treatment groups against the negative control.***, P ≤ 0.001; ns, not significant.

There was a statistically significant reduction between the groups treated with GA in all tested concentrations, except for the group treated with 2 times the MIC in the 24h biofilm (Fig 4). An improvement of GA activity on 48h biofilm at 2 times the MIC was unexpected, since the biofilm maturation occurs between 24 and 48h, when the complex structures of the biofilms are increased with all fungal cell types (hyphae and yeast) present and involved by a multilayered matrix [37]. Further investigation should be conducted in order to understand this phenomenon. One hypothesis could be based on the dynamics of biofilm formation. In the biofilm of 24h, both cellular and molecular features, as well as the water content, are different from 48h biofilm [38]. These differences can modulate the efficacy of GA. Moreover, although the mechanism of antifungal activity of GA was not fully understood, this molecule could act in the matrix compounds, which are quantitatively more present in the 48h biofilm [39].

In this present study the set value of MIC was 5mg/mL, considering the experiments with standard and clinical strains of *C*. *albicans*. The classic protocol of the National Committee for Clinical Laboratory (2008) used to obtain the MIC values of an antifungal agent evaluates mostly the susceptibility of the cells in suspension. This is not the case of the biofilms, which are attached to biotic or abiotic surfaces [40]. Due to its complexity, concentrations higher than the MIC are in general requested for biofilms [4, 41]. Alves *et al*. [42] have showed a GA antibiofilm activity for *C*. *albicans* at 5mg/mL, which was 32 times the MIC value found in that study. However, the authors have used only standard strains whereas in this present study clinical strains were also used. It is known that clinical strains are less susceptible to the antimicrobial agents [43]. Therefore, in this study, we have chosen to work with concentrations higher than the MIC.

Nonetheless, with 4 times the MIC (20 mg/mL) GA becomes insoluble in aqueous solvents, since the water solubility of GA is approximately 12 mg/mL or 70mM, as observed in the Fig 1 or in the literature [44]. Thanks to the complexation of the GA by HPβCD, which improved the GA solubility, it was possible to test 4 times the MIC. Interestingly, the treatments of 24 and 48h biofilms with GA/HPβCD spray-dried microparticle did not differ from the treatment with AmB, which is a molecule of reference for the treatment of fungal infections [45]. Therefore, GA/HPβCD spray-dried complexes showed a promising antibiofilm activity and could be another option for the therapy against *C*. *albicans*, mainly in the case of resistance to the available drugs.

The effect of HPβCD solvent control (180 mg/mL of HPβCD dissolved in RPMI) on *Candida albicans* biofilm was tested. The results did not show any difference between the group treatment (HPβCD solvent, without GA) and the control (only RPMI, without molecules), with P value equal to 0.5104 (t test). The counts of CFU/specimen for the control and treatment groups were, respectively, 1.4 ± 1.1 × 10^−6^ and 1.3 ±0.7 × 10^−6^. This concentration of 180 mg/ml was selected because 200 mg/mL of GA/HPβCD spray-dried complexes was used for antifungal activity evaluation, which is equivalent to 20 mg/mLof GA and 180 mg/mL of HPβCD.

#### Effect of GA/HPβCD for the treatment of oral candidiasis induced in rat model

**Fig 5:**
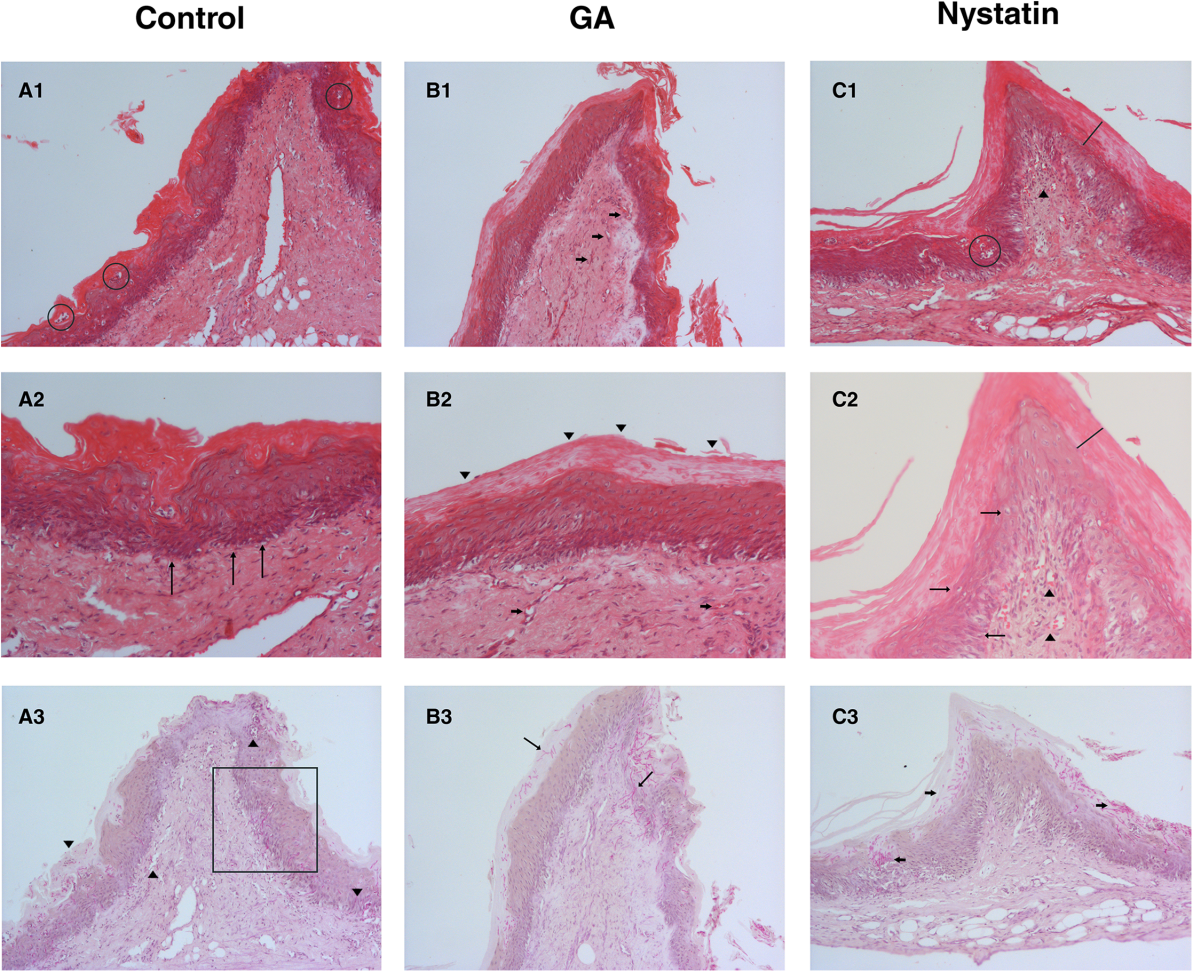
Antifungal activity on oral candidiasis in rats. Control Group (A), GA/HPβCD spray-dried microparticles group (B) and Nystatin group (C). (A1) Histologic sections stained by Hematoxylin and eosin (HE) method, 100X magnification, exhibiting hyperkeratosis, and micro-abscesses formation (circle) in the epithelium that is also acanthotic. The fibrous connective tissue has wide vessels surrounded by a moderate inflammatory infiltrate. (A2) PAS staining, 100X magnification, where *C*. *albicans* yeast and mostly hyphae invade the epithelium reaching the connective tissue (arrowheads). (A3) HE staining, 200X magnification. Inflammatory epithelial alterations as hyperkeratosis, epithelial stratification loss (square), and basal layer disorganization (arrows) are evident. (B1) Histologic sections stained by hematoxylin and eosin (HE) method, 100X magnification, exhibiting hyperkeratosis, and acanthosis of the epithelium that covers a hyper-vascularized (arrows) connective tissue. (B2) HE staining, 200X magnification. Reactive hyperkeratosis of the epithelium (arrowheads) was the only remarkable inflammatory induced alteration for the GA/HPβCD group. Some congest vessels can be seen in the connective tissue (arrows). (B3) PAS staining, 100X magnification, where *C*. *albicans* yeast and mostly hyphae invade the epithelium, some of them reaching the connective tissue (arrows). (C1) Histologic sections stained by hematoxylin and eosin (HE) method, 100X magnification, hyperkeratosis (line), micro-abscess formation (circle), and acanthosis of the epithelium that covers a hyper-vascularized (arrowheads) connective tissue can be seen. (C2) PAS staining, 100X magnification where large groups of *Candida albicans* yeast and hyphae can be noted. Though most of them are limited to the keratin layer, some invade the epithelium (arrows). (C3) HE staining, 200X magnification, where epithelial hyperkeratosis (line) is evident. We can see poor inflammatory alteration (arrows), as hydropic degeneration and duplication of the basal layer. Some congest vessels can be seen in the connective tissue (arrowheads) surrounded by moderate inflammatory infiltrate.

Fig 5 shows the effect of GA/HPβCD for the treatment of oral candidiasis induced in rat model.

The oral tissue was chosen for *in vivo* experiments due to the ability of *C*. *albicans* to produce oropharyngeal candidosis and to form biofilm over the tongue and oral mucosa [46]. Animals from the control group (treated with saline solution) exhibited invasive candidiasis evidenced in the PAS stained slides. Several *Candida* hyphae appear at the keratin and granular layers of the epithelium, some of them invading the squamous layer. Interestingly, some hyphae can be seen invading the connective tissue at punctual areas of the soft palate. According to the HE staining, areas of hyperkeratosis of the epithelium can be noticed, and also many epithelial alterations, such as duplication of the basal layer, increased nuclear-cytoplasmic ratio, enlarged cells, and hydropic degeneration in the squamous layer. Also, there are areas of intraepithelial abscess and acanthosis. The connective tissue, in general, is fibrous, hypervascularized and shows a moderate mononuclear infiltrate (Fig 5A1, 5A2 and 5A3).

**Fig 6:**
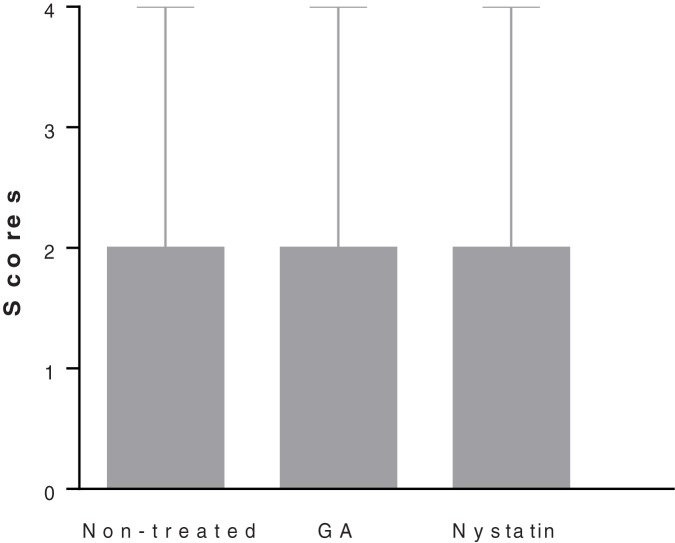
Analysis of hypha counting. Median with range of the attributed scores for each group after hypha counting.

*Candida* hyphae could be also noted in the treated groups, some of them invading the epithelium and there was no difference in the invasion scores among the groups (Fig 6). Future evaluation of other protocols, i.e. increasing the frequency of treatment, should be performed.

The HE staining shows that the GA/HPβCD spray-dried microparticles group had a mild inflammatory response with no intraepithelial abscess formation or cellular alterations. Only some mononuclear cells were spread in the connective tissue and there was a moderate subepithelial infiltrate (Fig 5B1, 5B2 and 5B3).

The nystatin group presented intraepithelial abscesses, hyperkeratosis, acanthoses, and no individual cell alteration. The connective tissue showed dispersed erythrocytes among the fibers, and moderate mononuclear infiltrate. The results of the nystatin therapy were thus histologically less effective than the GA/HPβCD spray-dried microparticles. Nonetheless, the epithelial and inflammatory responses were reduced for nystatin group compared to the control group (Fig 5C1, 5C2 and 5C3). Nystatin was used as positive control because this molecule is a reference for the treatment of oral candidiasis in clinical practice [47].

Similarly to nystatin (standard treatment), the GA/HPβCD spray-dried microparticles were able to reduce inflammatory response in the mucosae *in vivo*.

## Conclusions

This study showed an improvement of GA solubility after complexation with HPβCD. This finding is relevant since GA has demonstrated activity on *C*. *albicans* biofilm, which requires high concentrations to reduce its viability. On the other hand, the phase solubility studies showed that increased concentrations of βCD in the presence of an excess amount of GA produced insoluble complexes, probably due to the low water solubility of βCD. The measured percentage of GA present in GA/HPβCD was 10.8 ± 0.01%, which is in accordance with the theoretical prediction of the phase solubility study. Moreover the encapsulation efficiency was 100%. This high value avoids the waste of raw material. The DSC and SEM analysis suggested the formation of an inclusion complex between GA and HPβCD. Noticeable antibiofilm activity for spray-dried GA/HPβCD was shown. The *in vivo* result suggests that the GA/HPβCD inclusion complexes, similarly to nystatin, was not able to prevent fungal tissue invasion but reduced inflammatory response, though further steps are needed to consolidate their use in humans. This study can thus contribute to the development of new therapies against *C*. *albicans* and helps to solve the problem of drug resistance by fungal micro-organisms.

## Supporting information

S1 File Solubility diagram data. Supporting information file of Fig 1.
(XLSX)

S2 File DSC thermograms. Supporting information file of Fig 2.
(XLSX)

S3 File Biofilm 24 and 48 h data. Supporting information file of Fig 4.
(XLSX)

S4 File Hypha counting data. Supporting information file of Fig 6.
(XLSX)
